# 3D reconstruction of structures of hatched larva and young juvenile of the larvacean *Oikopleura dioica* using SBF-SEM

**DOI:** 10.1038/s41598-021-83706-y

**Published:** 2021-03-01

**Authors:** Hiroki Nishida, Nobuhiko Ohno, Federico Caicci, Lucia Manni

**Affiliations:** 1 Department of Biological Sciences, Graduate School of Science, Osaka University, 1-1 Machikaneyama-cho, Toyonaka, Osaka 560-0043 Japan; 2 Division of Ultrastructural Research, National Institute for Physiological Sciences, Okazaki, 444-8787 Japan; 3 Division of Histology and Cell Biology, Department of Anatomy, Jichi Medical University, Shimotsuke, Tochigi 329-0498 Japan; 4 Dipartimento di Biologia, Università degli Studi di Padova, Via U. Bassi 58/B, 35131 Padova, Italy

**Keywords:** Developmental biology, Zoology

## Abstract

The larvacean *Oikopleura dioica* is a planktonic chordate and an emerging model organism with a short life cycle of 5 days that belongs toTunicata (Urochordata), the sister clade of vertebrates. It is characterized by the rapid development of a tadpole-shaped body. Organ formation in the trunk proceeds within 7 h after the hatching of the tailbud larvae at 3 h after fertilization (hpf) and is completed at 10 hpf, giving rise to fully functional juveniles as miniature adult form. Serial block face scanning electron microscopy was used to acquire ~ 2000 serial transverse section images of a 3 hpf larva and a 10 hpf juvenile to characterize the structures and cellular composition of the trunk and organs using 3D images and movies. Germ cells were found to fuse and establish a central syncytial cell in the gonad as early as 10 hpf. Larval development gave rise to functional organs after several rounds of cell division through trunk morphogenesis. The feature would make *O. dioica* ideal for analyzing cellular behaviors during morphogenetic processes using live imaging. The detailed descriptions of the larvae and juveniles provided in this study can be utilized as the start and end points of organ morphogenesis in this rapidly developing organism.

## Introduction

The larvacean *Oikopleura dioica* is a planktonic chordate with a short life cycle of 5 days. It is a promising model organism that belongs to Tunicata (Urochordata), which is the sister clade of vertebrates^1^ and has various advantages for the analysis of the process of organ formation at a single cell level using live imaging, as has been demonstrated by studies of the development of specialized arrangements of epidermal cells to secrete the so-called “house”^2^ and of mouth formation^3^. Embryos and hatching larvae are small (ca. 100 µm) and are entirely transparent. Adult specimens are ca. 5 mm long with a tadpole shape, consisting of small number of cells (ca. 4000 cells) with a dorsal central nervous system, notochord, gill slits, endostyle, and postanal tail, all of which are chordate characteristics. Organ formation in the trunk occurs during 7 h after the hatching stage of tailbud larvae, which is at 3 h after fertilization (hpf). Organ formation is completed and fully functional juveniles are formed at 10 hpf, at which point they start feeding and are considered to be miniature adult forms. At this stage, the cell division of most somatic cells, except for those in the digestive organs, ceases and the cells start to increase their size via endoduplication, culminating in the development of sexually mature adults on the fifth day^4^. Although the stages of embryogenesis and cell lineages up to hatching at 3 hpf have been well described^1,5–8^, our knowledge of larval development and organ formation remains relatively limited.

Serial block face scanning electron microscopy (SBF-SEM) is a powerful tool used to acquire serial ultrathin sections and reconstruct 3D stuructures of cells, tissues, and small organisms. It collects large volumes of 3D information with resolutions at the electron microscopic level^9^. The resolution of the resulting images is very high (8 nm and 12,000 × 12,000 pixels, in this study). For example, SBF-SEM allows for the close visualization of mitochondrial cristae, as well as entire transverse sections of the body (96 × 96 µm, in this study). The small size of *O. dioica* larvae and juveniles allows them to be suitable for SBF-SEM observation and 3D reconstruction in order to investigate their body structures at the single-cell level.

In this study, the structures and cellular compositions of organs in 3 hpf larvae and 10 hpf juveniles are evaluated. The larval development of *O. dioica* occurs via trunk morphogenesis, starting from a simple cell mass and developing into functional organs over several rounds of cell division. The larvae are small and completely transparent. These are ideal features for the analysis of cellular behaviors during various morphogenetic processes using live imaging. Detailed descriptions of the structures and cellular compositions of the larvae and juveniles provide the start and end points of organ morphogenesis in this rapidly developing organism. The descriptions are also useful for interpreting the results of gene knockdown/knockout experiments using this organism, which evolved a body with extremely simplified morphology and small cell number.

## Methods

### Laboratory culture of *O. dioica*

*O. dioica* was obtained from a culture maintained in our laboratory, as previously described^10–12^. *O. dioica* is dioecious with a five-day life cycle at 20 °C. Naturally spawned eggs from isolated females were artificially fertilized with sperm. Ethical approval is not required for handling *O. dioica* according to the ethical and legal guidelines of Osaka University.

### Serial block face scanning electron microscopy (SBF-SEM)

Sample preparation and observation by SBF-SEM was performed as described in detail by Morita et al.^3^. Larvae at 3 hpf were fixed within 10 min of hatching. Juveniles were fixed at 10.5 hpf, 30 min after the tail shift^1^. In brief, the specimens were fixed with 2.5% glutaraldehyde and 2.0% paraformaldehyde, and post-fixed with 2% osmium tetroxide and 1.5% potassium ferrocyanide in 0.1 M sodium cacodylate buffer (pH 7.4) and 50% artificial seawater. The samples were then stained with 1% uranyl acetate in distilled water and 0.2 M lead aspartate before embedding in a Durcupan resin (Sigma-Aldrich).

SBF-SEM observations were performed according to Morita et al.^3^, using a Merlin scanning electron microscope (Carl Zeiss Microscopy, Jena, Germany) equipped with a 3View in-chamber ultramicrotome system (Gatan, Pleasanton, CA). The serial slice thickness was 70 nm. Images were recorded at an accelerating voltage of 1.3 kV and dwell time of 1.0 µs. The image size was 12,000 × 12,000 pixels, with a resolution of 8 nm, covering a total area of 96 × 96 µm. After 2 × 2 binning of the images to reduce noise, the image stack was automatically aligned using ‘Register Virtual Stack Slices’ in the Fiji/ImageJ software. For the 3 h larva, 1812 images were acquired and the total file size after binning was 68 GB. For the 10 h juvenile, 1967 images were acquired and the total file size was 74 GB.

### Image segmentation and 3D construction

Tissue structures were manually segmented in the image of each section and reconstructed into three dimensions (3D) using the Amira software package (ver. 5 and 6, FEI Visualization Science Group, Burlington, MA, USA; https://www.thermofisher.com/jp/en/home/industrial/electron-microscopy/electron-microscopy-instruments-workflow-solutions/3d-visualization-analysis-software/amira-life-sciences-biomedical.html) according to Morita et al.^3^. Amira was also used to generate figures and movie files. The centers of each nucleus were recorded, and nuclei were represented as arbitrarily sized small spheres in 3D reconstructions, using Microscopy Image Browser software (Electron Microscopy Unit, Institute of Biotechnology, University of Helsinki, Finland; http://mib.helsinki.fi/) to show positions and number of nuclei, and the data was exported in Amira-compatible data format.

### Ethics approval

Ethical approval and consent to participate were not required for this study.

## Results and discussion

### Structures of hatching larva (Fig. 1, Supplementary Movie S1-3)

**Figure 1:**
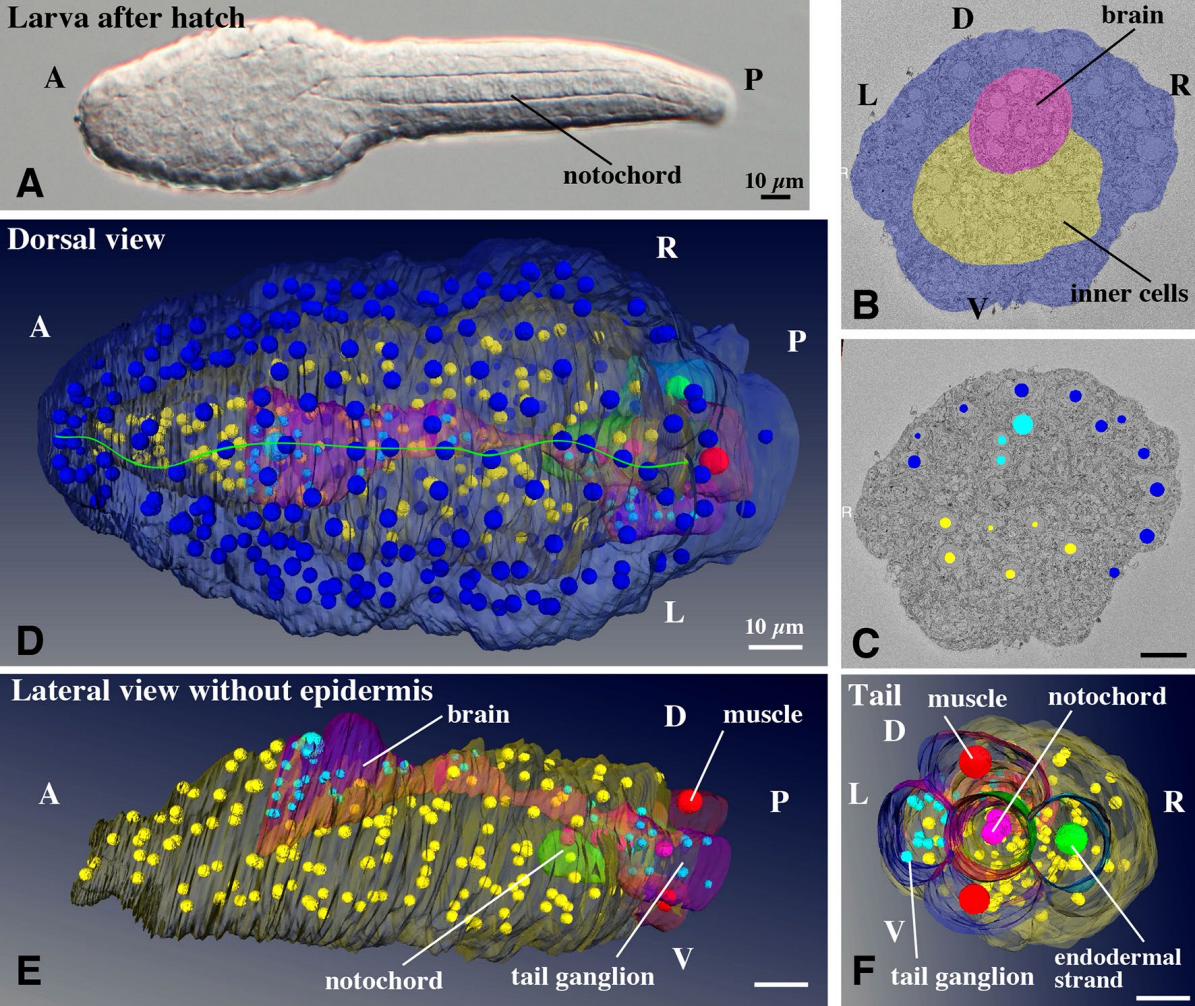
Structure and cellular organization of a hatched larva of *O. dioica.* (**A**) A larva just after hatching at 3 hpf. (**B**) Segmentation of tissue in a SBF-SEM section image. Cells facing the surface of the larva are shown in dark blue, and inner cells are shown in yellow. The central nervous system is shown in pink. Anterior view. See Supplementary Movie 1 for the entire series of serial sections. (**C**) The positions of nuclei of cells facing the surface of the larva are shown in dark blue, and those of inner cells are shown in yellow. The nuclei of the central nervous system are shown in light blue. See Supplementary Movie 1 for the entire series of serial sections. (**D**) 3D reconstruction of serial SBF-SEM sections of a hatched larva. Dorsal view. The full trunk and base of the tail are shown. The central nervous system is shown in pink with light blue nuclei. Green line indicates the dorsal midline. Note that the size and shape of nuclei do not reflect those in the sample and are represented by arbitrarily-sized spheres to show the position and number of nuclei. The nuclei of the surface cells are arranged in bilateral symmetry. See Supplementary Movie 2 for the rotating 3D image. (**E**) Lateral view without epidermis. See Supplementary Movie 3 for the rotating 3D image. (**F**) Posterior view of the tail. The tail ganglion is present on the left side. (**B**–**F**) The images were generated using the Amira software package (ver. 5 and 6). A, anterior; P, posterior; D, dorsal; V, ventral; L, left; R, right; Scale bars, 10 µm.

Tadpole shaped-larvae hatched at 3 hpf at 20 °C (Fig. 1A) and started intermittently beating their tail, where the notochord and muscles are present. In contrast, there was almost no recognizable structure inside the trunk region when observed under a light microscope.

A total of 1812 images of the trunk region were acquired every 70 nm with SBF-SEM, and thereafter segmented. Only three tissues were recognized in the trunk, epidermis, brain, and inner cells (Fig. 1B, Supplementary Movie S1 for the entire series of serial sections). At the base of the tail, muscles, notochord, tail ganglion, and endodermal strand were visible. To characterize the composition of the cells, the positions of the centers of each nucleus were recorded and nuclei were represented as small spheres in 3D space (Fig. 1C, Supplementary Movie S1). It is worth noting that the size and shape of the nuclei do not reflect those in the sample and are represented by arbitrarily sized spheres (in most cases, smaller than the real size) to show the position and number of nuclei. Figure 1D and Supplementary Movie S2 shows the 3D reconstruction of the segmented data. The epidermis cells, represented by positions of the dark blue nuclei, showed accurate bilateral symmetry along the dorsal midline (Fig. 1D, green line). In the ventral half, they were also bilaterally arranged (Supplementary Movie S2). The trunk epidermis of the juveniles and adults, called the oikoplastic epidermis, was comprised of elaborate cellular arrangements showing a complex bilateral pattern for the secretion of the so-called house, which is composed of extracellular components^2, 13,14^. This indicated that the bilateral arrangement of precursor cells of the oikoplastic epidermis was already attained by the hatching stage.

The inner structures are shown in Fig. 1E,F, and Supplementary Movie S3. A rudimentary central nervous system with a smooth outline (pink with light blue nuclei) was recognized in the serial sections. However, the anterior border was ambiguous. This may result from the larva being cut into transverse sections, or more likely, the anterior part of the brain is formed in later developmental stages. There was a single large cell in the dorsal part of the brain (pink dorsal bulge with large light blue nucleus). Stem cell-like cell divisions contribute to generating the anterior part of the brain in *Oikopleura*^2^; therefore, the large cell observed here is likely to represent the stem cell within the brain. The posterior part of the brain was connected to the tail ganglion via a dorsal cellular process with the dorsal cellular process going around the notochord towards the left side (Supplementary Movie S1), and connected to the tail ganglion, which was present on the left side of the tail base. Most of the inside of the trunk was filled with inner cells (yellow). A clear bilateral symmetry in the arrangement of inner cells, as well as that of the nervous system, was not observed. The cell count in the trunk region of the larva shown in Fig. 1A was 501 and is summarized in Table 1. Cell counts in a whole body in the previous study^6^ is also shown in Table 1 for comparison.

**Table 1:**
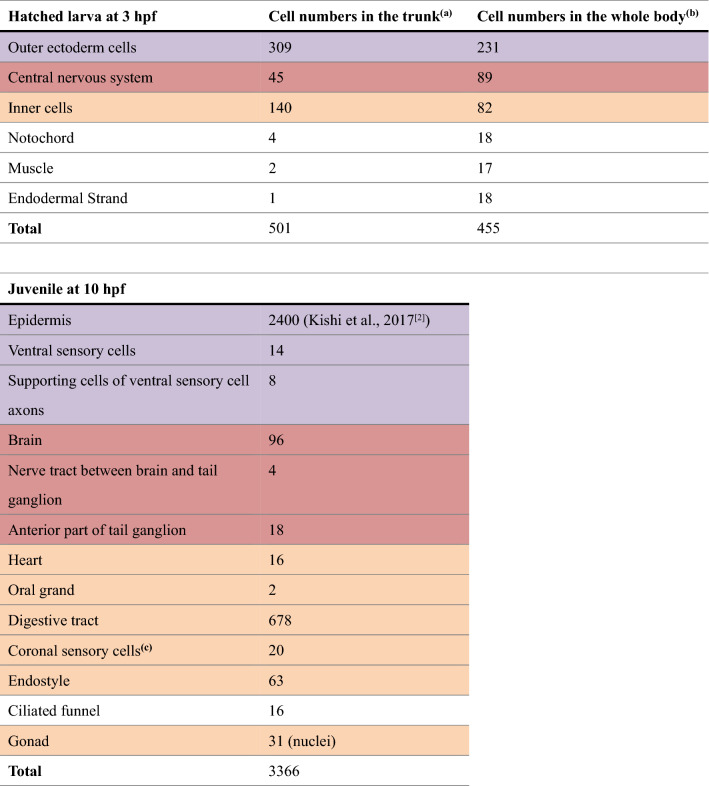
Cell counts in the larva and juvenile.

Different colors indicate possible progeny relationship between the larva and juvenile.

**Figure 2:**
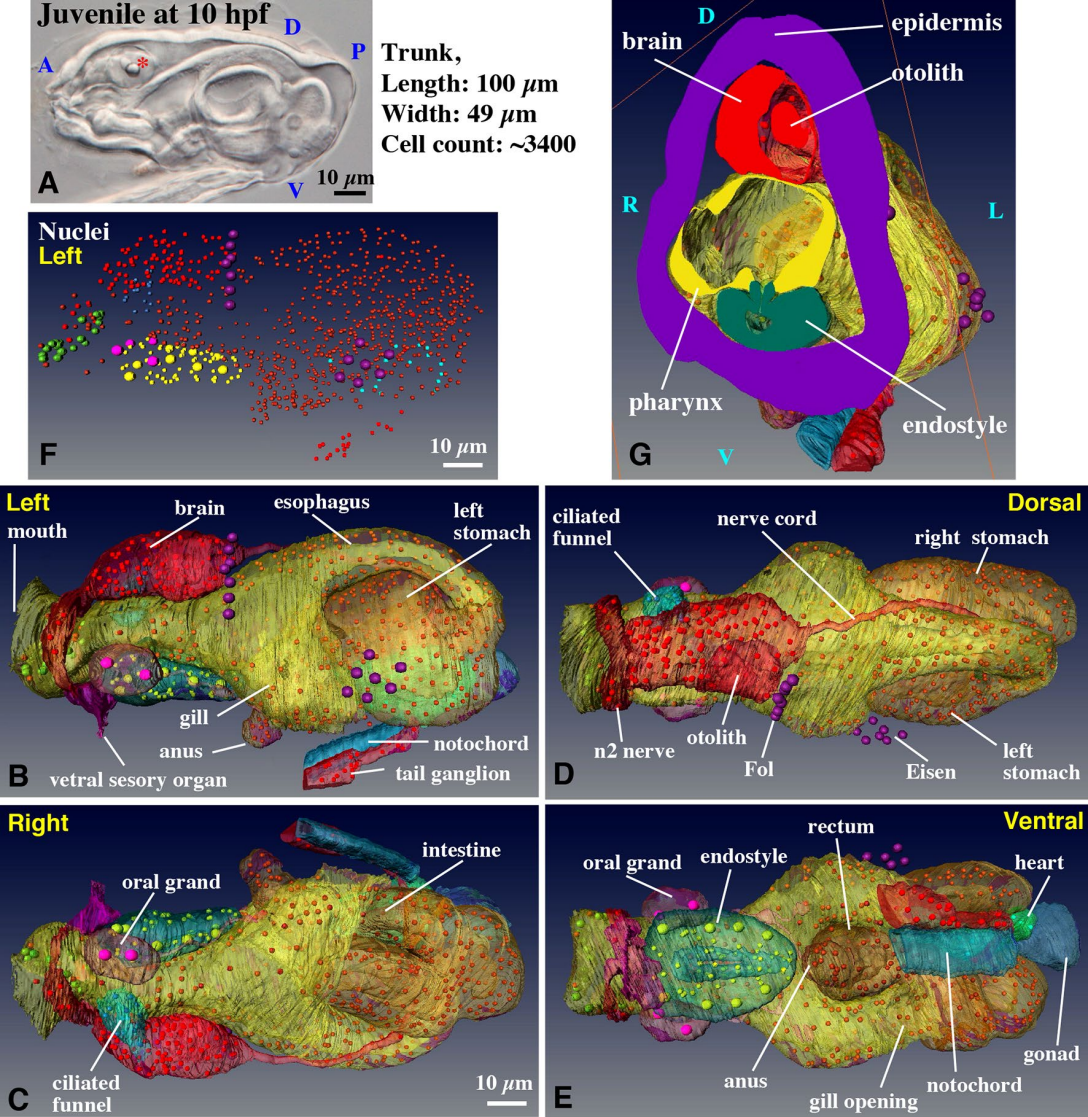
Structure and cellular organization of a young juvenile. Note that labeling order of photos in this panel is irregular. (**A**) A juvenile just after tail shift at 10 hpf. Most somatic cell divisions were completed by this stage, resulting in a body organization similar to that of adult animal. Organ formation was accomplished and the specimens started to feed. Otolith within the brain vesicle is indicated by asterisk. (**B**–**E**) 3D reconstruction of serial SBF-SEM sections. The full trunk and base of the tail are shown in the left, right, dorsal, and ventral views, respectively. The epidermis is removed. Only nuclei of giant Fol cells and giant Eisen cells on the left epidermis are shown, as positional landmarks. See Supplementary Movie 7 for the rotating 3D image. In (**E**), the heart (green) is covered by notochord and tail ganglion, thus most of the heart cannot be seen (see Fig. 4d and Supplementary Movie 13 for heart). The nuclei of the gonad in (**E**) is not shown (see Fig. 5 and Supplementary Movie 20 for gonad). (**F**) Positions of nuclei. The number of nuclei shown here counts 921 without gonad (31 nuclei). See Supplementary Movie 8 for the rotating 3D image. (**G**) Cross-section of the trunk showing the cavities of each organ and the thickness of the epithelia. Otolith within the sensory vesicle of the brain is visible on the dorsal side. See Supplementary Movie 9 for the entire series of transverse sections. (**B**–**G**) The images were generated using the Amira software package (ver. 5 and 6). A, anterior; P, posterior; D. dorsal; V, ventral; L, left; R, right. Scale bars, 10 µm.

^a^Cell numbers were counted using data of the present study. Cells were counted in the trunk region that is shown in Fig. 1D and Supplementary Movie S2 for the larva, and Fig. 2B,F and Supplementary Movie S7 for the juvenile. The larva is post-hatching larva.

^b^Cell numbers in the whole body including the tail region were counted using the cell lineage tree in Stach et al.^6^. In this case, the larva is pre-hatching larva. Note that cell numbers of outer ectoderm cells and inner cells are much lower than those of post-hatching larva because these cells continue dividing during hatching time.

^c^Coronal sensory cells are ciliated mechanosensory cells that are present in the oral region, and mediate ciliary reversal in the gills^3^. They are represented as green nuclei in the anterior region in Supplementary movie S7 and 8.

Previously, transmission electron microscopy (TEM) has been used to observe hatching larvae^6,15^. Especially, Stach et al., (2008) made a complete series of sections of a newly hatched larvae, observed it with TEM, segmented tissue boundaries, marked positions of nuclei in the nervous system, epidermis, notochord and muscle, and reconstructed 3D anatomy of the trunk and tail [mainly shown in the Supplementary Figures of the reference 6]. The observations of Stach et al. and us coincide well. They also distinguished three kind of cells, epidermis cells, cells in nervous system, and inner cells (designated endoderm in the anterior region and undifferentiated meso-/endoderm in the posterior region although the boundary of these two was not clearly defined). We were not able to determine anterior border of the central nervous system based on a smooth outline of the tissue as mentioned above. In contrast, Stach et al. demarcated the anterior border based on cellular positions and smaller cell size. Therefore, cell counts in the brain region is 46 in Stach et al. and 28 in the present study. Another difference is that they observed small cavities in the center of both the nervous system and endoderm. We did not see such cavities in our sample (Supplementary Movie S1). In this study, larvae were fixed within 10 min of hatching. It could be that their sample was fixed at a bit advanced stage after hatching.

### Overall structures of juvenile (Fig. 2, Supplementary Movie S4–9)

A larva that completed morphogenesis at 10 hpf was observed by SBF-SEM (Fig. 2A). The tail shifted ventrally, after which juveniles expand the house and start feeding^1^. Every organ, with the exception of the gonad, is already functional at this stage. Thus, juveniles at 10 hpf can be considered miniature adults. Most of the cell divisions of the epidermis cease by this stage and cells enter the endocycling stage; DNA is replicated without cell division. In contrast, the cells of the digestive organ continue to divide to some extent after this stage^4^.

The cell counts in the trunk region of the juvenile are summarized in Table 1. The total cell count was 3366. The cell count of the epidermis appears to be slightly underestimated in this table, since Kishi et al.^2^ only counted the cell number of the oikoplastic epidermis region, which does not contain epidermal cells that cover the gonad region, although the number of epidermal cells that cover the gonad region is small and this does not much affect the total cell count. During the 7 h between the 3 hpf and 10 hpf stages, the epidermis cells divide approximately 3 times on average^2^. The cell number of the central nervous system increased 2.6-fold, while the number of inner cells increased 6.0-fold, giving rise to various endodermal and mesodermal organs. Thus, the larval development of *O. dioica* represents the process of morphogenesis from a simple cell mass into functional organs, involving only a few rounds of cell division. This is an advantageous feature of *O. dioica* as a model organism for the analysis of cellular behaviors during various morphogenetic processes using live imaging, as has been previously shown during the development of the oikoplastic epidermis and mouth in *O. dioica*^2,3^.

A total of 1967 images of the trunk region were acquired every 70 nm. Supplementary Movies S4 and S5 show the segmentation of organs and nuclei for the entire series of serial sections. The organs and nuclei were consistently colored through the study. The nuclei of the epidermis were not segmented. The segmented data of transverse sections (Movie S4) were converted to show the dorsal to ventral frontal sequence (Movie S5). The segmented data were used for 3D reconstruction as shown in Movie S6. Overall 3D structures of the trunk region are shown without epidermis (Fig. 2B–E, Movie S7), while the positions of all nuclei were plotted to show the cellular organization of the organs (Fig. 2F, Movie S8). The ventral sensory organ (Fig. 2B, purple) and nuclei of giant Fol cells and giant Eisen cells (Fig. 2D, purple nuclei) on the left side are shown as landmarks in the epidermis. In these figures and movies, it is difficult to recognize cavities inside each organ and the thickness of the epithelia. Therefore, transverse sections were also displayed in the 3D representation of the trunk (Movie S9). All epithelia of *O. dioica* juveniles are single-layered, similar to those in adults.

The overall morphological organizations of the adults and juveniles of *O. dioica* have been reviewed in Fenaux^16^ and Nishida^1^, respectively. The adult structures have also been previously observed by scanning electron microscopy^17^. In brief, the juvenile has a brain (red in 3D reconstructions in the present study) in the dorsal trunk region. The digestive tract shows a conspicuous left–right asymmetry (e.g. the esophagus is connected to the left stomach; see also Fig. 4B–E) and is depicted in yellow. Seawater enters the mouth and exits the bilateral gill openings, which are situated ventrally. In the pharynx, food particles are entangled in mucus secreted from the endostyle (green). The food particle-laden mucus is transported through the dorsal esophagus, left stomach, right stomach, intestine, and rectum. Fecal pellets are discharged via the anus, which is situated in the mid-ventral region. A ciliated funnel (light blue) is present on the right side of the pharynx, connecting its cavity to the pharynx, and its blind tip is attached to the brain. Juveniles have oral glands (pink) on both sides of the pharynx, the heart between the left and right stomachs (green, most part is hidden by other organs in Fig. 2B–E), and gonad (dark blue) in the posterior-most region. In the following sections, each organ will be described in detail in the following order: ectodermal, mesodermal, endodermal, and reproductive organs.

The structure and development of the oikoplastic epidermis is well described in the literature^2,14^, as well as those of the mouth region^3,18^. Therefore, we excluded these from this study except for the Fol domain and gill opening. In addition to the Supplementary Movies, we have provided an interactive 3D PDF file, which enables the viewing of the 3D reconstruction of *O. dioica* juveniles in any direction. Each organ can be hidden or rotated using the computer mouse. The interactive 3D PDF file (123 MB) (see the supplementary file for the legends and how to use it) is available in the Dryad repository (https://doi.org/10.5061/dryad.hmgqnk9dw). The datasets of the entire SBF-SEM serial section images are also available in the Dryad repository (see Data Availability section).

### Fol domain (Fig. 3A–D, Supplementary Movie S10)

**Figure 3:**
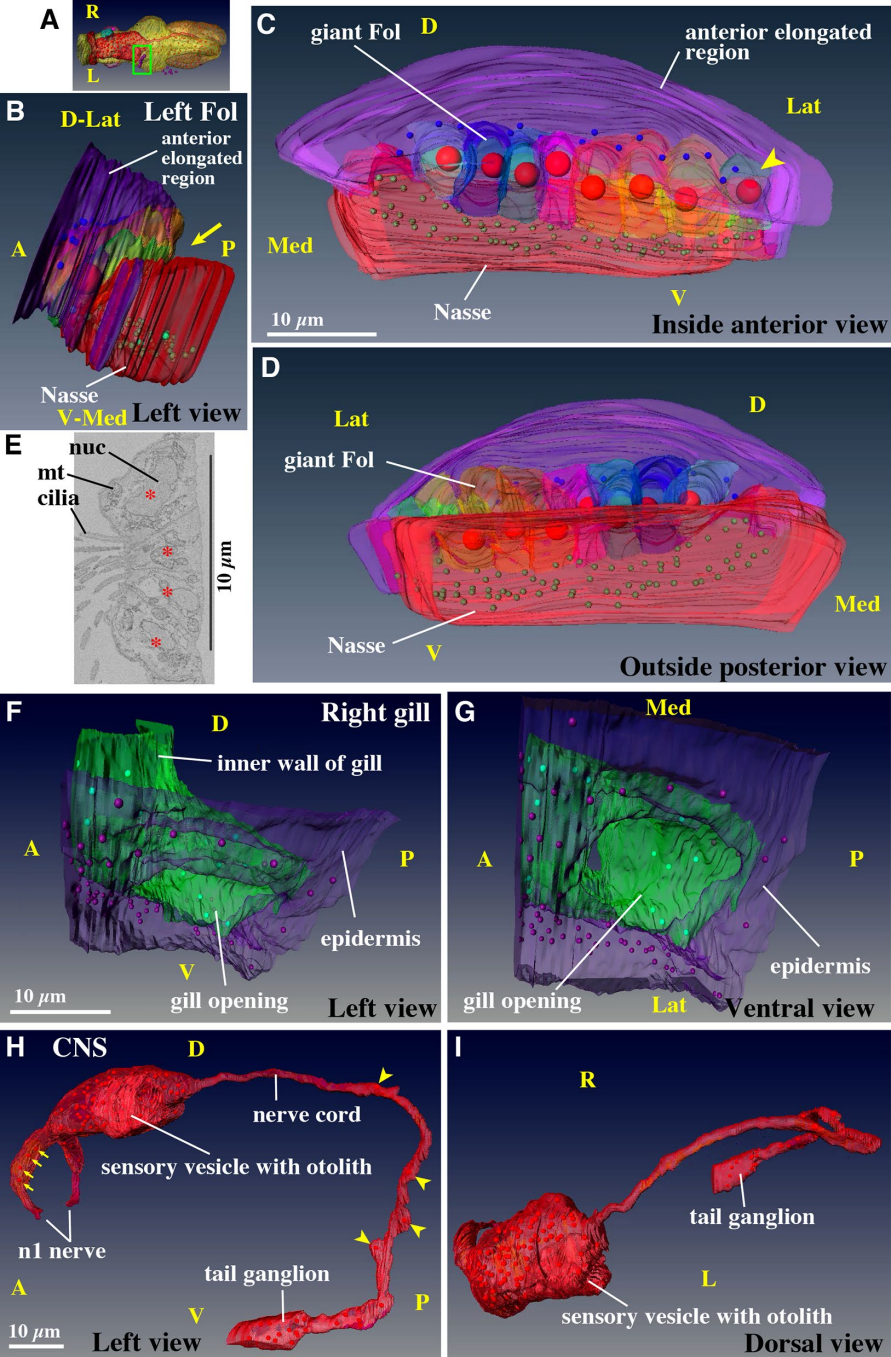
Ectodermal derivatives. (**A**–**D**) Left Fol domain in the oikoplastic epidermis. Giant Fol cells (various colors) are indicated by large red nuclei in (**B**–**D**) (also shown by purple nuclei in Fig. 2B,D). Each giant Fol cell is segmented. Only the posterior part of the anterior elongated region (purple) is shown. The arrow indicates the groove between the giant Fol cells and the Nasse cells (red). The arrowhead points to the 8th giant Fol cell. See Supplementary Movie 10 for the rotating 3D image. (**A**) Position of the left Fol domain in the trunk (green box in dorsal view). Purple nuclei correspond to nuclei of the giant Fol cells (see also Fig. 2D). (**B**) Left-ventral side view. (**C**) Anterior view, which is observed from the left side of (**B**). (**D**) Posterior view, which is observed from the right side of (**B**). (**E**) Ciliated cells in the inner wall of the gill. Four cells have cilia that are extended towards inside of the gill. The center of each cell is marked by asterisk. Ciliated cells form a band encircling the gill. (**F**,** G**) Detailed 3D construction of the right gill opening (the position is shown in Fig. 2E), viewed form the left side (i.e. from the mid line). The epidermis (purple) and inner wall of gill (green) are connected at the opening. See Supplementary Movie 11 for the rotating 3D image. (**H**,** I**) Central nervous system. The brain contains 96 cells. The tail ganglion is located at the base of the tail. The arrows indicate four supporting cells in the n2 nerve. The arrowheads point to four cells in the nerve cord. See Supplementary Movie 12 for the rotating 3D image. (**A**–**D**,** F**–**I**) The images were generated using the Amira software package (ver. 5 and 6). D. dorsal; V, ventral; Med, medial; Lat, lateral; nuc, nucleus; mt, mitochondria. Scale bars, 10 µm.

The oikoplastic epidermis is specialized in the secretion of house, in which the animal lives. The Fol domain in the epidermis is conspicuous and has a complex structure and unique cell composition of various cell sizes. Bilateral Fol domains secrete the food-concentration filters of the house^13,19^ and protrude slightly outwards. The left-side Fol domain was segmented and used to construct a 3D image. This domain consists of a single-layered epithelium comprised of roughly three-sized cells: cells of the anterior elongated region (only the posterior part of the anterior elongated region is shown in Fig. 3B–D), giant Fol cells, and Nasse cells^2^. Nasse cells are aligned in three rows containing approximately 30 cells each, although the lateral edges of Nasse domain were not able to precisely determined. The cells have a rectangular shape, and are perfectly arranged into a latticework. The position of the nuclei of the giant Fol cells in the entire trunk is shown by the purple nuclei in Figs. 2B,D and 3A.

In the 3D image (Fig. 3B–D, Supplementary Movie S10), the anterior elongated region with mid-sized cells (purple cells with blue nuclei) dorso-laterally covered the giant Fol cells (various colored cells with large red nuclei). The giant Fol cells were aligned in a single row and ventrally flanked by many small-sized Nasse cells (red cells with green nuclei). There was a grove between the giant Fol cells and Nasse cells (Fig. 3B, yellow arrow). The grooves are also clearly visible in Supplementary Movies S4 and S5. The nuclei in this domain were always located on the basal side of the cells. We segmented each giant Fol cell in various colors with red nuclei. The apical side of the giant Fol cells faced outwards of the body and in the posterior direction (Fig. 3D), while the basal side faced inwards (Fig. 3C). The spatial arrangement of the three-sized cells is likely to play a role in the secretion and building of a complex food-concentration filter with a fine mesh. However, how the food-concentration filter is formed has not yet been elucidated. In adult, the number of giant Fol cells is definitely seven^2^. However, upon 3D construction, additional single cells, which showed similar features to giant Fol cells, albeit slightly smaller, were present in the lateral part (Fig. 3C, arrowhead). It is possible that this cell will be lost after larval development^2^. It has been reported that four epidermis precursor cells of hatched larva divide once to generate the eight giant Fol cells, while three rows of the Nasse cells on one side are derived from more than ten precursor cells by two or mostly three rounds of lateral cell divisions^2^.

### Gill opening (Fig. 3E–G, Supplementary Movie S11)

The water current is driven by ciliary movements in the gill. There was a ring of ciliated cells surrounding the gill cavity (Supplementary Movie S4). The ring was four-cells wide (Fig. 3E: The center of each cell is marked by an asterisk.), as reported in adults^20^. We segmented the right-side gill opening (Fig. 2E) in more detail. The gill opening (Fig. 3F,G, Movie S11: The ciliated ring is not included in these figures as it locates deeper in the gill cavity as seen in Supplementary Movie S4.) had a simple structure in which the epidermis (purple) and inner wall of gill (green) were connected. The inner wall of the gill was very thin and consisted of a few flat cells (green nuclei).

### Central nervous system (Fig. 3H,I, Supplementary Movie S12)

The structure of the nervous system in adults is described in the literature^21,22^, and matched the observations of juveniles in the present study. The cell count of the brain was 96, including a huge otolith cell (Fig. 2A, asterisk and 2G, Movie S9)^23^ embedded in a sensory vesicle on the left side. The trunk nerve cord extended from the posterior side of the brain (Fig. 3H,I, Movie S12). It extended posteriorly, and turned ventrally around the posterior part of the bridge between the left and right stomachs, eventually turning in the anterior direction and connecting to the tail ganglion (also called caudal ganglion^24^). The trunk nerve cord appeared as a bundle of approximately ten axons; however, four cell bodies with nuclei were present along the nerve cord (Fig. 3H, yellow arrowheads).

In terms of the peripheral nervous system, bilateral n1 nerve bundles (the nomenclature of Olsson et al.^21^) were created from 14 mono-ciliated ventral sensory organ cells (Fig. 2B, Movie S9, pink)^25^ and innervated the anterior brain. The pair of bundles was associated with four supporting cells aligned in a line on each side (also shown in red in Fig. 3H, yellow arrows). Bilateral n2 nerves emanated from the sensory neurons in the brain, consisting of dendrites that received information from the 20 oral mechanosensory cells (called coronal cells) of the circumoral ring (Fig. 2E, Movie S7 and S8, green nuclei), as observed in the adults^18^. Their structures have been described in detail by Morita et al.^3^. The n3 nerves that innervate the ciliated ring in the gill and ventral epidermis were also recognized (not shown in Figures as the n3 nerve is very thin consisting of a single axon). However, in contrast to adults^21^, we did not find the axons connecting to the dorsal epidermis. These axons may have been overlooked, or may form at a later stage of juvenile development.

### Heart (Fig. 4A, Supplementary Movie S13, 14)

**Figure 4:**
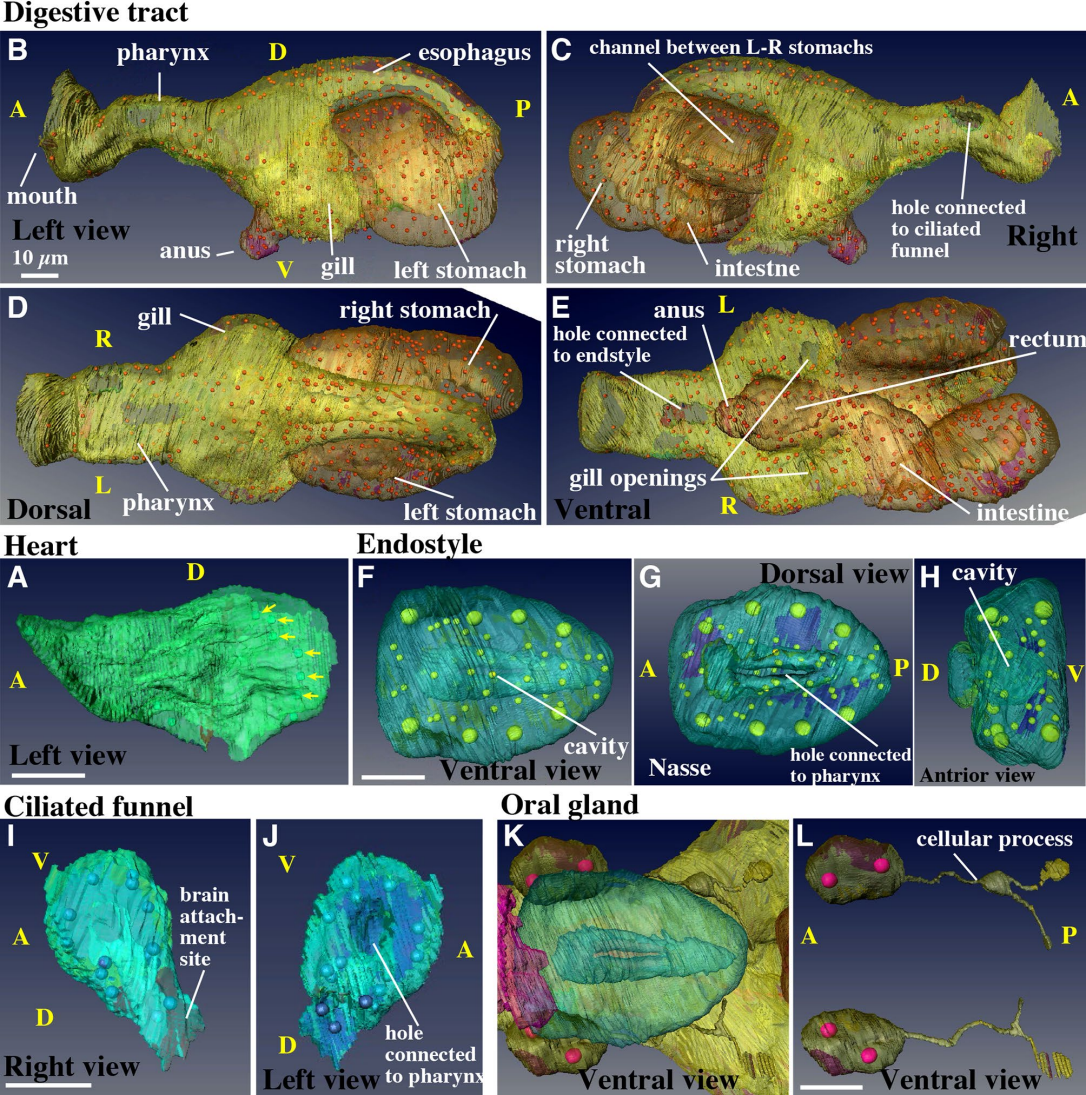
Mesodermal and endodermal derivatives*.* Note that labeling order of photos in this panel is irregular. (**A**) Heart. The muscle layer of the heart is shown in the left view. The arrows indicate the nuclei of the cardiac muscle cells. See Supplementary Movie 13. (**B–E**) Digestive tract. Seawater is filtered out through the gills, while food is passed down through the mouth, pharynx, esophagus, left stomach, the channel between the L-R stomach, the right stomach, intestine, rectum, and anus. See Supplementary Movie 15. (**F–H**) Endostyle. The cavity is connected to the pharynx shown in (**E**). See Supplementary Movie 16. (**I**,**J**) Ciliated funnel. The cavity is connected to the pharynx shown in **C**. See Supplementary Movie 17. (**K**,**L**) A pair of oral gland cells on both sides of the pharynx. See Supplementary Movie 18. The images were generated using the Amira software package (ver. 5 and 6). Scale bars, 10 µm.

The heart is beating as early as in the 10 hpf juveniles. The blood (hemolymph) circulates in the interspace between the organs of the trunk and tail (open blood–vascular system), pumped by the beating of the heart. *O. dioica* has no blood cells in the blood fluid. The heart of ascidians is tubular, and the muscle layer forms an inner tube that is connected to the outer pericardium epithelium on one side of the tube along the long axis, resulting in a double-layered tube^26^. A pericardial cavity is present between the two tubes. Thus in other words, the muscle cells are part of the pericardium, and the heart is a longitudinal fold along the pericardium. In contrast, the heart of the *O. dioica* adults does not fold like those in ascidians and is a simple sac^16^, which is consistent with our observations. The heart was present on the right side of the left stomach (Movie S4 and S9, green), was elongated in the anterior–posterior direction, and bent slightly towards the left stomach (Fig. 4A, Movie S13). In the serial sections (Movie S14), since the right wall of the sac was thin, it was considered to be pericardial epithelium. The left wall was thicker, with six long muscle cells aligned in the dorsal to ventral direction, resulting in a ribbed structure along the anterior–posterior axis. The nuclei were present in the posterior region (Fig. 4A, yellow arrows). According to the literature, the left wall is comprised of a cardiac muscle layer, although it was difficult to recognize muscle fibers in the SBF-SEM images. Thus, while in ascidians, the muscle layer is rolled up to make a tube, it is a flat wall in *O. dioica*. Blood flow is thought to be generated in the narrow space between the cardiac muscle layer and the left stomach.

### Digestive tract (Fig. 4B–E, Supplementary Movie S15)

There are four regions where endodermal and ectodermal single-layered epithelia meet to form an opening to the outside of the body: the mouth, two gill openings on the left and right sides, and the anus^1, 17,27^. In the digestive tract, two additional holes were observed, which were connected to the ciliated funnel and endostyle on the right-dorsal and ventral sides of the pharynx, respectively (Fig. 4C and E). The stomach had two lobes, the left and right stomach, connected by a bridge (Movie S9). The esophagus was connected to the posterior part of the left stomach (Fig. 4B and D). Food particles were transferred to the right stomach and exited from the anterior-ventral part of the right stomach into the intestine (Fig. 4C and E). Thus, a conspicuous left–right asymmetry was observed in the arrangement of the digestive organs. There was no visceral muscle. Food particles trapped by mucus in the pharynx are carried by the cilia of the alimentary tract cells. Although the cilia within the digestive tract were not segmented, they are visible as gray matter within the tract in Movie S4. The cell count of the entire digestive tract was 678. The number of cells in each digestive organ could not be counted because the boundaries between the organs were hard to determine precisely. The entire digestive tract was made of a single-layered epithelium. Generally, the thickness of the epithelium was thinner in the anterior parts and thicker in the posterior parts (Movie S9). The ultrastructures of the cells of the digestive organ in adults have been observed in previous studies^27^.

### Endostyle (Fig. 4F–H, Supplementary Movie S16).

The endostyle of tunicates is regarded as a homolog of the thyroid of vertebrates. Although they accumulate iodine^28^, the main function of the endostyles of ascidians and larvaceans is to secrete mucus into the pharynx to trap food particles mixed with seawater introduced via the mouth. The endostyle lies ventrally to the pharynx, and is comprised of a pouch made of a single-layered epithelium. In the present study, it was found to consist of 63 cells and showed a bilaterally symmetrical cellular arrangement. The cavity was connected to the pharynx through a slit on its dorsal side (Fig. 4F–H, Movie S16). The structure of the endostyle of adults has been described in the literature^13,28–30^. Several cell types are distinguished: from the ventral side, ventromedial cells, ciliated cells, glandular cells (also called gland cells and lateral arm endostyle cells), connecting cells, corridor cells. These cell types were distinguished as early as in the 10 hpf juvenile (Movie S4, green). In particular, large glandular cells, namely mucus-secreting cells, were easily recognized due to their large cell bodies and nuclei. Four glandular cells were found on each side of the 10 hpf juvenile (Fig. 4F,G, represented by large nuclei). Glandular cells have been previously found to enter the endocycle phase and increase the DNA amount up to 194-fold^31^. The number of glandular cells increases during juvenile development, and new glandular cells are supplied from the small cells in the posterior region^31^. The number of cells gradually increases from 4 to 16–20 on one side.

### Ciliated funnel (Fig. 4I,J, Supplementary Movie S17)

The ciliated funnel had a conical shape, was comprised of 16 cells, and had an inner cavity. It was present on the right side of the pharynx and brain, with the cavity connected to the pharyngeal cavity (Figs. 2C and 4I,J, Movie S17). The cavity of the ciliated funnel was filled with cilia, which are motile^17^. The left side of the cone was closely attached to the brain. The tip of the cone was blind. Although it is not clear whether the ciliated funnel is derived from the brain or digestive tract during larval development, Delsman^32^ reported that it protrudes from the brain. This could parallel what has been reported in ascidians, in which the ciliated duct rudiment (the larval neurohypophyseal duct) originates from the anterior neural plate of embryos^33^, which is thought to be the source of the postmetamorphic brain^34^. Function of the ciliated funnel is controversial and has yet to be fully elucidated. A previous study reported on the ultrastructures of ciliated funnel cells in adult specimens^35^.

### Oral gland (Fig. 4K,L, Supplementary Movie S18)

A pair of oral gland cells was present on both sides of the pharynx (Fig. 4K), which contained two nuclei in a single cell. Although the physiological function of the oral gland cells is still under discussion^36–38^, it has been suggested to be involved in endogenous bioluminescence in this organism. Bioluminescence has been exclusively observed in the family Oikopleuridae. Among Oikopleuridae, species with oral glands show bioluminescence, whereas species lacking oral glands do not^36^. Luminescence is exerted by small particles (called inclusion bodies restricted to bioluminescent species) in the expanded house and house rudiment upon stimulation^37^. The oral gland itself does not show bioluminescence. It has been suggested that oral gland cells have a fine cellular process piercing the epidermis and that the tip outside the epidermis is sequentially cut off and attached to house rudiment as the inclusion bodies^38^. However, we did not observe this cellular process, despite careful observation. Instead, a fine cellular process was present posteriorly and branched into two parts (Fig. 4L, Movie S18). One of the two branches terminated as a flat sheet attached to the ventral side of the gill duct. However, this type of structure has never been reported and it is difficult to infer the function of this process solely by its shape.

The oral gland cells were found to have two nuclei, consistent with the observations in adults^31^. The amount of DNA in single nuclei in adults increases by 1248-fold after endoduplication^31^. The formation of the oral gland during larval development is a peculiar process^39^. An oral gland precursor cell has four nuclei and migrates from the posterior region to the anterior region of the trunk. It becomes U-shaped upon reaching the midline endostyle, which settles on the migratory pathway. The oral gland precursor cell is eventually pulled apart at the bottom of the U shape at 6 hpf into left and right cells, each cell inheriting two nuclei. Each cell has two nuclei until the end of the life, just repeating endoduplication.

### Gonad (Fig. 5, Supplementary Movie S19, 20)

**Figure 5:**
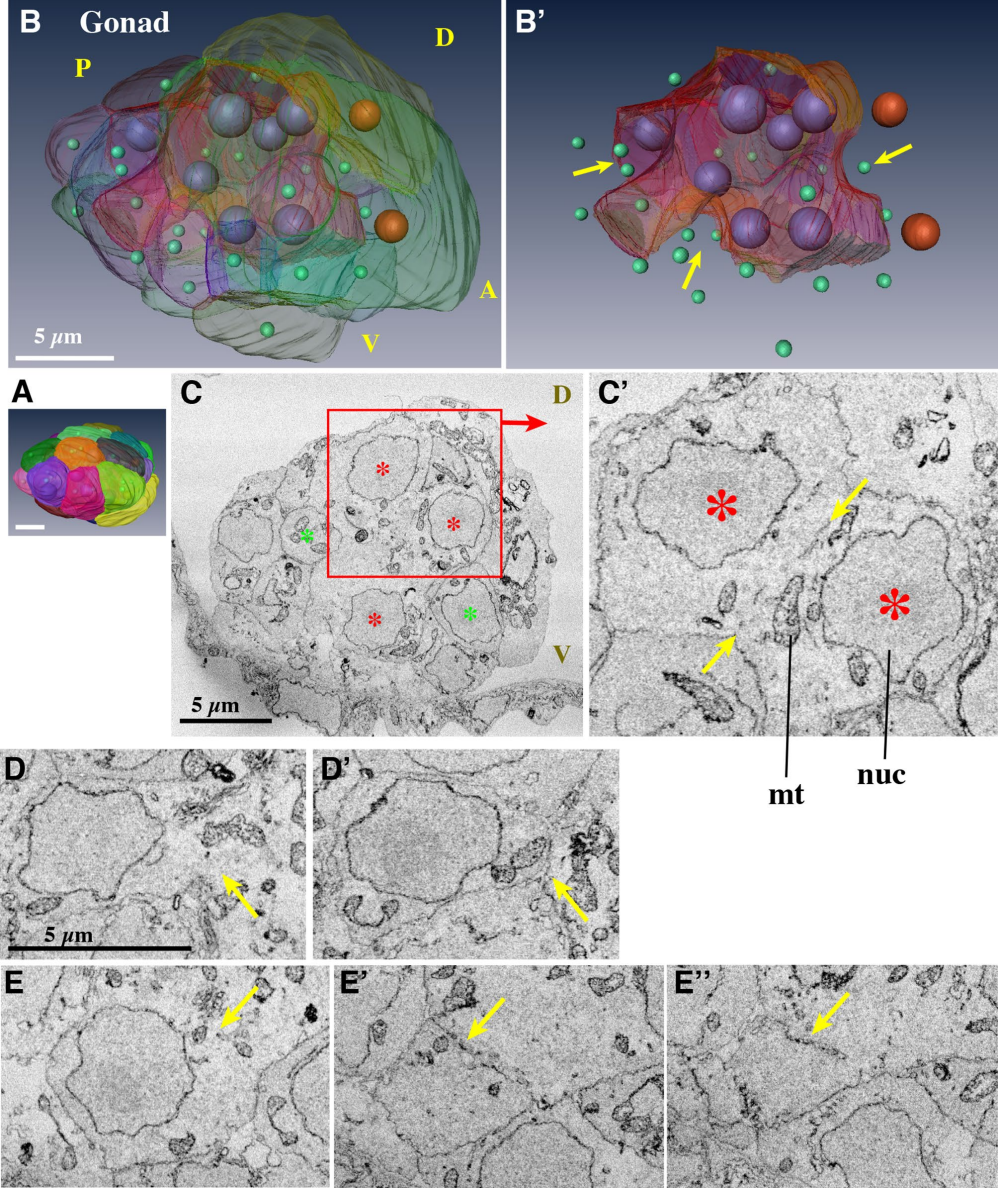
Gonad. (**A**) Surface view. Anterior is to the right. (**B**) Each cell is represented in a distinct transparent color. Seven large purple nuclei are shown within the central syncytium (red cell). Two large brown nuclei denote large surface cells (green and yellow cells). Other cells that have a single nucleus are indicated by small green nuclei. Note that the size and shape of the nuclei do not reflect those in the sample and are presented as arbitrarily-sized spheres. See Supplementary Movie 19 and 20. (**B’**) The central syncytium is shown in red. The arrows indicate grooves. (**C**) Section of the central syncytium. Three nuclei (asterisks) share cytoplasm. (**C’**) Another section in the z-stack of the two nuclei that are shown in the rectangle in **C**. Putative cell membrane remnants are visible between the two arrows. (**D**, **D’**) A cell that is fusing. The cell is fused to the central syncytium in **D**, but in another section, there is clear cell membrane between it and the adjacent cell in **D’** (arrows). (**E**, **E’**, **E’’**) Another example of a fusing cell. Three sections of the cell. The cell is fused to the central syncytium in **E**. The cell membrane is fragmented in (**E’**) (arrow). The membrane is continuous, but lost on the right side in (**E’’**). (**A**,**B’**) The images were generated using the Amira software package (ver. 5 and 6). Scale bars, 5 µm.

The gonad was present in the most posterior part of the trunk (Fig. 2E, light blue). Each cell in the gonad was segmented using different colors to show the cellular organization of the gonad at 10 hpf (Fig. 5A,B). *O. dioica* is the only dioecious species among the larvaceans. In the developing ovary of day 2–5 animals, several hundred pro-oocytes with meiotic nuclei are linked via ring canals, namely plasma bridges, to a common cytoplasm shared by several thousand polyploid nurse nuclei^40–42^. A single syncytial body with a common cytoplasm is called a coenocyst. Testis development has been previously observed using transmission electron microscopy^43^. The developing testis also forms syncytium, but without nurse nuclei. However, it is not known when and how the gonad syncytium forms. The present observation suggests that gonad syncytium formation already starts as early as in 10 hpf juveniles by the cell fusion of germ cells, although it is not possible to discern males or females at this stage.

There are two primordial germ cells in the posterior region of the trunk at hatching^6^. We attempted to identify the primordial germ cells in the SBF-SEM images of the 3 hpf larvae, but it was not possible. No special cells, for example, containing germ plasm, were observed. The gonad of the 10 hpf juvenile consisted of 25 cells and 31 nuclei (Table 1, Movie 19 for serial sections). Among these, 7 nuclei were present within a central syncytial cell (Fig. 5B,B’,C, and Movie 20, large red cell with large purple nuclei). Sixteen cells faced the surface of the gonad (Fig. 5A), two of which were large (yellow and green cells with large brown nuclei Fig. 5A), while eight cells that were not fused were present inside the gonad.

Upon careful observation, the syncytium appeared to form via cell fusion. First, in the middle, between the two syncytial nuclei, possible remnants of the plasma membrane were occasionally observed (Fig. 5C’, flanked by two yellow arrows). Second, some cells seemed to undergo fusion. For example, the cell in Fig. 5D is connected to the common cytoplasm (arrow), but is entirely surrounded by the cell membrane in another section (Fig. 5D’). Similarly, the cell in Fig. 5E is connected to the common cytoplasm. The cell membrane is present in other sections, but the membrane is apparently fragmented (Fig. 5E’,E’’, arrows). These findings indicate that the cell fusion of germ cells was likely to have started, but was still under way in the 10 hpf juvenile. In this regard, 8 cells that were not fused inside the gonad and embedded in grooves (Fig. 5B’, arrows) may fuse to the central syncytium after this stage. After completion of cell fusions, multiple nuclear divisions will follow without cytokinesis. Cells that face the surface of the gonad showed no indication of cell fusion. These cells may develop into follicle cells that cover the male and female gonad syncytium^43,44^.

All the data in this report are from a single hatched larva and a single juvenile. There could be possible variations in cellular numbers and composition in organs, although the variations between individuals are expected to be small. At least, morphological traits of this animal are highly invariant^16,17^. In addition, cell lineages during embryogenesis has been known to be invariant^6–8^, as well as spatial arrangement of epidermis cells in the oikoplastic epidermis^2^. During larval development, cell numbers and behaviors are stereotyped for cells that show long distance migration^39^ and cells that are involved in formation of the oral region^3^. Therefore, even the data from a single animal would be valuable for future studies.

*O. dioica* evolved into a simplified chordate species with small genome size, short life cycle, and small body that consists of small number of cells, while still keeping the basic body plan of chordates. Thus, *O. dioica* is a useful model animal for studying ontogenetic and phylogenetic processes in chordates.

## Conclusion

Due to its short life cycle of five days, *O. dioica* progresses rapidly from the larval to juvenile stages of development. In fact, the morphogenesis of various trunk organs is completed at 7 h of larval development. In this study, we evaluated the structural and cellular composition of all organs in a 3 hpf larva and a 10 hpf juvenile in detail. Every organ except for the gonad is already functional in10 hpf juveniles, which are considered miniature adults. Thus, the larval development of *O. dioica* represents morphogenesis from a simple cell mass to functional organs, involving only a few rounds of cell division. Given that the larvae are small and completely transparent it provides advantages for the analysis of cellular behaviors during various morphogenetic processes using live imaging. The descriptions provided in this study could be utilized as the start and end points of organ morphogenesis in this rapidly developing organism. The detailed descriptions of the juvenile morphology provided herein could also be useful for interpreting gene knockdown and knockout experiments using this organism^45,46^, which has evolved a body with an extremely simplified morphology and a small number of cells.

## Supplementary Information

Supplementary Information.

Supplementary Video 1.

Supplementary Video 2.

Supplementary Video 3.

Supplementary Video 4.

Supplementary Video 5.

Supplementary Video 6.

Supplementary Video 7.

Supplementary Video 8.

Supplementary Video 9.

Supplementary Video 10.

Supplementary Video 11.

Supplementary Video 12.

Supplementary Video 13.

Supplementary Video 14.

Supplementary Video 15.

Supplementary Video 16.

Supplementary Video 17.

Supplementary Video 18.

Supplementary Video 19.

Supplementary Video 20.

## Data Availability

The datasets of SBF-SEM serial section images and an interactive 3D PDF file supporting the conclusions of this article are available in the Dryad repository. Images of the juvenile (42 GB): https://doi.org/10.5061/dryad.d51c5b012. Images of the larva (36 GB): https://doi.org/10.5061/dryad.dbrv15dzh. The interactive 3D PDF file (123 MB) and Supplementary Movie files (S1-20, 470 MB): https://doi.org/10.5061/dryad.hmgqnk9dw. The AMIRA datasets and files generated during the current study are available from the corresponding author upon request.

## References

[1] Nishida H (2008). Development of the appendicularian *Oikopleura dioica*: culture, genome, and cell lineages. Dev. Growth Differ..

[2] Kishi K, Hayashi M, Onuma TA, Nishida H (2017). Patterning and morphogenesis of the intricate but stereotyped oikoplastic epidermis of the appendicularian, *Oikopleura dioica*. Dev. Biol..

[3] Morita R, Onuma TA, Manni L, Ohno N, Nishida H (2020). Mouth opening is mediated by separation of dorsal and ventral daughter cells of the lip precursor cells in the larvacean, *Oikopleura dioica*. Dev. Genes Evol..

[4] Ganot P, Thompson EM (2002). Patterning through differential endoreduplication in epithelial organogenesis of the chordate, *Oikopleura dioica*. Dev. Biol..

[5] Delsman HC (1910). Beiträge zur Entwicklungsgeschichte von *Oikopleura dioica*. Verh. Rijksinst Onderz. Zee..

[6] Stach T, Winter J, Bouquet JM, Chourrout D, Schnabel R (2008). Embryology of a planktonic tunicate reveals traces of sessility. Proc. Natl. Acad. Sci. USA.

[7] Fujii S, Nishio T, Nishida H (2008). Cleavage pattern, gastrulation, and neurulation in the appendicularian, *Oikopleura dioica*. Dev. Genes Evol..

[8] Stach T, Anselmi C (2015). High-precision morphology: bifocal 4D-microscopy enables the comparison of detailed cell lineages of two chordate species separated for more than 525 million years. BMC Biol..

[9] Kremer A, Lippens S, Bartunkova S, Asselbergh B, Blanpain C (2015). Developing 3D SEM in a broad biological context. J. Microsc..

[10] Bouquet JM, Spriet E, Troedsson C, Otterå H, Chourrout D, Thompson EM (2009). Culture optimization for the emergent zooplanktonic model organism *Oikopleura dioica*. J. Plankton Res..

[11] Omotezako T, Nishino A, Onuma TA, Nishida H (2013). RNA interference in the appendicularian *Oikopleura dioica* reveals the function of the *Brachyury* gene. Dev. Genes Evol..

[12] Martí-Solans J, Ferrández-Roldán A, Godoy-Marín H, Badia-Ramentol J, Torres-Aguila NP, Rodríguez-Marí A, Bouquet JM, Chourrout D, Thompson EM, Albalat R, Cañestro C (2015). *Oikopleura dioica* culturing made easy: a low-cost facility for an emerging animal model in EvoDevo. Genesis.

[13] Fenaux R (1986). The house of *Oikopleura dioica* (Tunicata, Appendicularia): structure and functions. Zoomorphology.

[14] Thompson EM, Kallesøe T, Spada F (2001). Diverse genes expressed in distinct regions of the trunk epithelium define a monolayer cellular template for construction of the Oikopleurid house. Dev. Biol..

[15] Nakashima K, Nishino A, Hirose E (2011). Forming a tough shell via an intracellular matrix and cellular junctions in the tail epidermis of *Oikopleura dioica* (Chordata: Tunicata: Appendicularia). Naturwissenschaften.

[16] Fenaux, R. Anatomy and functional morphology of the Appendicularia. In *The Biology of Pelagic Tunicates* (ed. Bone, Q), 55–80. Oxford University Press (1998). ISBN: 0-19-854024-8.

[17] Onuma TA, Isobe M, Nishida H (2017). Internal and external morphology of adults of the appendicularian, *Oikopleura dioica*: an SEM study. Cell. Tissue Res..

[18] Rigon F, Stach T, Caicci F, Gasparini F, Burighel P, Manni L (2013). Evolutionary diversification of secondary mechanoreceptor cells in tunicata. BMC Evol. Biol..

[19] Flood, P. R. & Deibel, D. The appendicularian house. In *The Biology of Pelagic Tunicates* (ed. Bone, Q), 105–124. Oxford University Press: Oxford (1998). ISBN: 0-19-854024-8.

[20] Martinucci DB, Dallai R, Burighel P, Casagrande L (1992). Ciliary specializations in branchial stigmatal cells of protochordates. Tissue Cell.

[21] Olsson R, Holmberg K, Lilliemarck Y (1990). Fine structure of the brain and brain nerves of *Oikopleura dioica* (Urochordata, Appendicularia). Zoomorphology.

[22] Bone, Q. Nervous system, sense organs, and excitable epithelia. In *The Biology of Pelagic Tunicates* (ed. Bone, Q), 55–80. Oxford University Press (1998). ISBN: 0-19-854024-8.

[23] Holmberg K (1984). A transmission electron microscopic investigation of the sensory vesicle in the brain of *Oikopleura dioica* (Appendicularia). Zoomorphology.

[24] Holmberg K, Olson R (1984). The origin of Reissner’s fibre in an appendicularian, *Oikopleura dioica*. Vidensk. Maddr Dansk Naturh. Foren..

[25] Bollner T, Holmberg K, Olson R (1986). A rostral sensory mechanism in *Oikopleura dioica* (Appendicularia). Acta Zoologica (Stockh.).

[26] Burighel, P. & Cloney, R. A. Urocordata: Ascidiacea. In: *Microscopy Anatomy of Invertebrates, Volume 15: Hemichordata, Chaetognatha and the Invertebrates Chordates.* (eds. Harrison R. and Ruppert E.), 221–347. Wiley-Liss, New-York (1997). ISBN: 0471561223.

[27] Burighel P, Brena C, Martinucci GB, Cima F (2001). Gut ultrastructure of the appendicularian *Oikopleura dioica* (Tunicata). Invertebrate Biol..

[28] Fredriksson G, Ofverholm T, Ericson LE (1985). Ultrastructural demonstration of iodine binding and peroxidase activity in the endostyle of *Oikopleura dioica* (Appendicularia). Gen. Comp. Endocrinol..

[29] Olsson R (1965). The cytology of the endostyle of *Oikopleura dioica*. Ann. N. Y. Acad. Sci..

[30] Cañestro C, Bassham S, Postlethwait JH (2008). Evolution of the thyroid: anterior-posterior regionalization of the *Oikopleura* endostyle revealed by Otx, Pax2/5/8, and Hox1 expression. Dev. Dyn..

[31] Troedsson C, Ganot P, Bouquet JM, Aksnes DL, Thompson EM (2007). Endostyle cell recruitment as a frame of reference for development and growth in the Urochordate *Oikopleura dioica*. Biol. Bull..

[32] Delsman HC (1912). Weitere Beobachtungen über die Entwicklung von *Oikopleura dioica*. Tijdschr Ned. Dierk. Ver. (Ser. 2).

[33] Imai JH, Meinertzhagen IA (2007). Neurons of the ascidian larval nervous system in *Ciona intestinalis*: I. Central nervous system. J. Comp. Neurol..

[34] Manni, L. & Pennati, R. Tunicata. In *Structure and Evolution of Invertebrate Nervous Systems* (eds. Schmidt-Rhaesa, A., Harzsch, S., and Purschke, G.) Chapter 53, 699–718. Oxford University Press (2016). ISBN: 9780199682201.

[35] Holmberg K (1982). The ciliated brain duct of *Oikopleura dioica* (Tunicata, Appendicularia). Acta Zoologica (Stockh.).

[36] Galt CP, Grober MS, Sykes PF (1985). Taxonomic correlates of bioluminescence among appendicularians (Urochordata: Larvacea). Biol. Bull..

[37] Galt CP, Sykes PF (1983). Sites of bioluminescence in the appendicularians *Oikopleura dioica* and *O. labradoriensis* (Urochordata: Larvacea). Marine Biol..

[38] Fredriksson G, Olsson R (1981). The oral gland cells of *Oikopleura dioica* (Tunicata Appendicularia). Acta Zool..

[39] Kishi K, Onuma TA, Nishida H (2014). Long-distance cell migration during larval development in the appendicularian, *Oikopleura dioica*. Dev. Biol..

[40] Ganot P, Kallesøe T, Thompson EM (2007). The cytoskeleton organizes germ nuclei with divergent fates and asynchronous cycles in a common cytoplasm during oogenesis in the chordate *Oikopleura*. Dev. Biol..

[41] Ganot P, Bouquet JM, Kallesøe T, Thompson EM (2007). The *Oikopleura* coenocyst, a unique chordate germ cell permitting rapid, extensive modulation of oocyte production. Dev. Biol..

[42] Ganot P, Moosmann-Schulmeister A, Thompson EM (2008). Oocyte selection is concurrent with meiosis resumption in the coenocystic oogenesis of *Oikopleura*. Dev. Biol..

[43] Cima F (2019). Spermatogenesis as a tool for staging gonad development in the gonochoric appendicularian *Oikopleura dioica* Fol 1872. Dev. Biol..

[44] Ganot P, Bouquet JM, Thompson EM (2006). Comparative organization of follicle, accessory cells and spawning anlagen in dynamic semelparous clutch manipulators, the urochordate Oikopleuridae. Biol. Cell..

[45] Omotezako T, Onuma TA, Nishida H (2015). DNA interference: DNA-induced gene silencing in the appendicularian *Oikopleura dioica*. Proc. R. Soc. B.

[46] Deng W, Henriet S, Chourrout D (2018). Prevalence of mutation-prone microhomology-mediated end joining in achordate lacking the c-NHEJ DNA repair pathway. Curr. Biol..

