# Supplementary material for: 3D reconstruction of structures of hatched larva and young juvenile of the larvacean Oikopleura dioica using SBF-SEM
Source: Sci Rep. 2021 Mar 1;11:4833. doi: 10.1038/s41598-021-83706-y (PMC7921577; doi:10.1038/s41598-021-83706-y)
Supplement: Supplementary file 1 — Supplementary Information. [file 41598_2021_83706_MOESM1_ESM.pdf]

Supplementary Information

3D reconstruction of structures of hatched larva and young juvenile of the larvacean *Oikopleura dioica* using SBF-SEM

Hiroki Nishida[1,*], Nobuhiko Ohno[2,3], Federico Caicci[4], Lucia Manni[4]

[1]Department of Biological Sciences, Graduate School of Science, Osaka University, Toyonaka, Osaka 560-0043, Japan
[2]Division of Ultrastructural Research, National Institute for Physiological Sciences, Okazaki 444-8787, Japan
[3]Division of Histology and Cell Biology, Department of Anatomy, Jichi Medical University, Shimotsuke, Tochigi 329-0498, Japan
[4]Dipartimento di Biologia, Università degli Studi di Padova, via U. Bassi 58/B, I-35121 Padova, Italy

***Corresponding author:** Hiroki Nishida, PhD
Department of Biological Sciences, Graduate School of Science, Osaka University
1-1 Machikaneyama-cho, Toyonaka, Osaka 560-0043, Japan
Tel: +81-6-6850-5472; Fax: +81-6-6850-5472
E-mail address: hnishida@bio.sci.osaka-u.ac.jp

Supplementary Materials

Supplementary PDF 1. Interactive 3D PDF file showing the structure of a 10 h juvenile (123 MB).

This PDF file enables the viewing of *O. dioica* juveniles in 3D in any desired direction. The organs can be hidden or rotated using the mouse. This PDF file is opened and activated as described in Adobe Acrobat User Guide (https://helpx.adobe.com/acrobat/using/displaying-3d-models-pdfs.html). Click Toggle "Model Tree button" with three small rectangles in the tool bar. Then, in the left panel, select the organs and nuclei of interest. Select lighting as "daylight". Select the solid or transparency mode. The interactive 3D PDF file is available in the Dryad repository (https://doi.org/10.5061/dryad.hmgqnk9dw).

Supplementary Movie S1. Segmentation of the SBF-SEM serial section images of a hatched larva. Related to Fig. 1B and C.

(The first half) Anterior view. The movie starts from the anterior side. The cells facing the surface of the larva are shown in dark blue, and inner cells are highlighted in yellow. The central nervous system is shown in pink. (The second half) Nuclear positions. The nuclei of the central nervous system are shown in light blue. The orientation and scale bars are as indicated in Fig. 1B and C.

Supplementary Movie S2. 3D movie of a hatched larva. Related to Fig. 1D.

The full trunk and base of the tail are shown. The movie starts from the dorsal view. Note that the size and shape of nuclei do not reflect those in the sample and are presented as arbitrarily-sized spheres to show the position and number of nuclei. The scale bar is as indicated in Fig. 1D.

Supplementary Movie S3. 3D movie of a hatched larva without epidermis. Related to Fig. 1E and F.

The full trunk and base of the tail are shown. The movie starts from the dorsal view.

Supplementary Movie S4. Segmentation of the SBF-SEM serial sections of a juvenile.

(The first half) Anterior view with the dorsal side up. The movie starts from the anterior side. Each organ is shown in distinct colors. The epidermis is shown in purple. The other colors correspond to those indicated in Fig. 2. The cilia within the digestive tract were not segmented, and are visible as gray matter within the tract. The sections slightly tilt along the left-right axis. (The second half) Nuclear positions.

Supplementary Movie S5. Segmentation of the juvenile: dorsal view.

A total of 1820 transverse sections were converted to show the frontal sections. (The first half)

Dorsal view. Anterior is to the left. The movie starts from the dorsal side. Each organ is shown in distinct colors. The epidermis is shown in purple. The other colors correspond to those indicated in Fig. 2. (The second half) Nuclear positions.

Supplementary Movie S6. 3D reconstruction of the transverse sections of a juvenile.
High-resolution images of 1820 transverse sections obtained with SBF-SEM were reconstructed into a 3D organization using Amira software.

Supplementary Movie S7. 3D rotation of inside juvenile structures. Related to Fig. 2B-E.
The full trunk and base of the tail are shown. The movie starts from the left view. The juvenile can be viewed from different angles. Note that the size and shape of nuclei do not reflect those in the sample and are presented as arbitrarily-sized spheres to show the position and number of nuclei. The scale bar is as indicated in Fig. 2C.

Supplementary Movie S8. 3D movie of nuclei of juvenile. Related to Fig. 2F.
Each position of the nuclei can be viewed in 3D space. The nuclei of the epidermis are not shown. The number of nuclei shown here is 921. The scale bar is as indicated in Fig. 2F.

Supplementary Movie S9. Entire series of transverse sections of the juvenile trunk. Related to Fig. 2G.
The cavity of each organ and the thickness of the epithelia are clearly seen in this movie.

Supplementary Movie S10. 3D movie of the left Fol domain. Related to Fig. 3A-D.
The movie starts from the left-ventral view.

Supplementary Movie S11. 3D movie of the right gill. Related to Fig. 3F and G.
The movie starts from the left view. A large purple nucleus indicates the position of a giant Eisen cell.

Supplementary Movie S12. 3D movie of the central nervous system. Related to Fig. 3H and I.
At the beginning of the movie, the position of the central nervous system is shown in the trunk. The movie starts from the left view.

Supplementary Movie S13. 3D movie of the heart. Related to Fig. 4A.
The movie starts from the left view, showing the muscle-layer side.

Supplementary Movie S14. Entire series of transverse sections of the heart. Related to Fig. 4A.
Anterior view with the dorsal side up. The right side of the heart is shown to the left. The movie starts from the anterior side. The muscle layer faces the left stomach (large organ on the

right of the images).

Supplementary Movie S15. 3D movie of the digestive tract. Related to Fig. 4B-E.

Supplementary Movie S16. 3D movie of the endostyle. Related to Fig. 4F-H.
The movie starts from ventral view.

Supplementary Movie S17. 3D movie of the ciliated funnel. Related to Fig. 4I and J
The movie starts from the right view with the dorsal side down.

Supplementary Movie S18. 3D movie of the oral gland. Related to Fig. 4K and L.
Movie starts from ventral view. Each cell has two nuclei and a cellular process.

Supplementary Movie S19. Entire series of transverse sections of the gonad. Related to Fig. 5.
Anterior view with the dorsal side up. The movie starts from the anterior side. Each cell is shown in a distinct color.

Supplementary Movie S20. 3D movie of the gonad. Related to Fig. 5B.
The movie starts from the left view. The anterior is to the left. Seven large blue nuclei are shown within the central syncytium (red cell). The two large brown nuclei denote large surface cells (green and yellow cells). Other cells with a single nucleus are indicated by small green nuclei.
